# CD62L^dim^ Neutrophils Specifically Migrate to the Lung and Participate in the Formation of the Pre-Metastatic Niche of Breast Cancer

**DOI:** 10.3389/fonc.2020.540484

**Published:** 2020-10-15

**Authors:** Zhen Wang, Chenghui Yang, Lili Li, Zhigang Zhang, Jun Pan, Ke Su, Wuzhen Chen, Jinfan Li, Fuming Qiu, Jian Huang

**Affiliations:** ^1^Key Laboratory of Tumor Microenvironment and Immune Therapy of Zhejiang Province, The Second Affiliated Hospital, Zhejiang University School of Medicine, Hangzhou, China; ^2^Cancer Institute, The Second Affiliated Hospital, Zhejiang University School of Medicine, Hangzhou, China; ^3^Department of Breast Surgery, The Second Affiliated Hospital, Zhejiang University School of Medicine, Hangzhou, China; ^4^Department of Oncology, The Second Affiliated Hospital, Zhejiang University School of Medicine, Hangzhou, China; ^5^Department of Gynecology, The Second Affiliated Hospital, Zhejiang University School of Medicine, Hangzhou, China; ^6^Department of Pathology, The Second Affiliated Hospital, Zhejiang University School of Medicine, Hangzhou, China

**Keywords:** breast cancer, lung metastasis, pre-metastatic niche, neutrophil, CD62L, migration, apoptosis

## Abstract

Lung metastasis is one of the leading causes of death in patients with breast cancer. The mechanism of tumor metastasis remains controversial. Recently, the formation of a pre-metastatic niche has been considered a key factor contributing to breast cancer metastasis, which might also explain the tendency of organ metastasis. Our study initially re-examined the critical time of the niche formation and simultaneously detected a novel subset of neutrophils, CD62L^dim^ neutrophils, which had not previously been reported in tumor metastasis; the number of these cells progressively increased during breast cancer progression and was closely related to the formation of the pre-metastatic niche. Furthermore, we explored the mechanism of their aggregation in the pre-metastatic niche in the lung and found that they were specifically chemoattracted by the CXCL12-CXCR4 signaling pathway. Compared to the CD62L^hi^ neutrophils, CD62L^dim^ neutrophils exhibited stronger adhesion and increased survival. The results provide new insights into the subsequent targeted treatment of breast cancer metastasis.

## Introduction

Breast cancer is currently one of the most common malignant tumors in women, and its mortality ranks third among malignancies in females (1). The most common cause of death is distant metastasis, which is the terminus of a series of tumor events. First, tumor cells from the primary tumor invade the surrounding tissues and enter the circulatory system. Only 0.01% of the tumor cells successfully settle in distant organs after leaving the primary tumor, despite its uncomplicated process (2). Afterwards, the tumor cells proliferate, leading to the establishment of metastasis. Importantly, the organ in which metastasis occurs is not randomly selected but is at least partially guided by the primary tumor. Substantial controversy still exists regarding the mechanism of tumor directional metastasis (3–5). In recent years, the formation of a pre-metastatic niche has attracted increasing attention (6–10). The pre-metastatic niche modifies the environment thereby promoting cancer cells to be seeded in specific organs, and hence the tumor cells dynamically interact with the surrounding environment to establish metastases (11, 12). The formation of the niche is a key factor contributing to tumor metastasis (13, 14).

Niche formation is presumed to require nutrients, extracellular matrix and immune cells. Metabolites promote the survival and migration of tumor cells (12). The extracellular matrix accelerates the attachment of tumor cells and activates the intracellular signaling pathways to improve their metastatic ability (15, 16). Meanwhile, a large number of prometastatic immune cells are enriched in the niche environment, which actively regulates the microenvironment by suppressing immune surveillance and immune killing functions (17–21).

Previous studies have considered neutrophils to be a crucial immune population regulating the formation of the pre-metastatic niche (22–25) because they maintain the characteristics of stem cancer cells through leukotriene signaling pathways (23) and build the immunosuppressive microenvironment by inhibiting the function of CD8+ T cells and natural killer (NK) cells (22, 26). In addition to the presence of different subsets of neutrophils, such as N1/N2 or low-density neutrophils/high-density neutrophils (LDN/HDN) in tumor tissues (27, 28), the heterogeneity of neutrophils has also gradually been recognized in tumor, such as antigen presenting cell-like (29), SiglecF^hi^ (30), CD13^+^ (31), CD177^+^ (32), c-MET^+^ (33), PD-L1^+^ (34), IL-17^+^ (35), iNOS^+^ (36). In the pre-metastatic niche, however, neutrophil heterogeneity remains unclear. An investigation of the factors contributing to the aggregation of different subsets of neutrophils in the lung is important and will subsequently facilitate the development of strategies to block the metastasis of tumor cells to the lung.

In the present study, the number of CD62L^dim^ neutrophils, which have not been studied in tumor metastasis, was increased significantly during the formation of the pre-metastatic niche. Further functional studies revealed that these neutrophils were specifically chemoattracted to the lung tissue by the CXCL12-CXCR4 signaling pathway and compared to other neutrophils, CD62L^dim^ neutrophils exhibited stronger adhesion and increased survival. The findings of this study provide a reference for further intervention research and clinical treatments targeting this cellular subset.

## Materials and Methods

### Cell Lines

The 4T1 cell line was provided by the Shanghai Institute of Cell Biology of the Chinese Academy of Science (SIBS, Shanghai, China). Cell lines were cultured in RPMI-1640 medium supplemented with 10% FBS (Gibco), 1:100 penicillin–streptomycin (Gibco) and 2 mmol/L Glutamine (Sigma-Aldrich). All cells were incubated in an incubator at a constant temperature of 37°C with a 5% CO_2_ atmosphere.

### Mice

Wild-type BALB/c female mice were purchased from Slaccas (Shanghai, China) and were fed in the Zhejiang Chinese Medical University Laboratory Animal Research Center. Six- to eight-week-old mice were randomly assigned to different groups. Mice were anesthetized with an intraperitoneal (*i.p.*) injection of 0.8% pentobarbital and 100 μL of the cell suspension (4T1 cells, 1x10^6^ cells/mL) were implanted in the right fourth mammary fat pad. MMTV-PyMT mice were a kind gift from Professor Qiyang Shou (Zhejiang Chinese Medical University). All female mice were genetically identified, and transgenic mice were selected for subsequent studies. All animal procedures were approved by the Ethical Review Committee of the Second Affiliated Hospital of Zhejiang University School of Medicine.

### Primary Tumor Resection

One or two weeks after 4T1 tumor inoculation *in situ*, mice were anesthetized with 0.8% pentobarbital (*i.p.*) then restrained on a thermostatic heating plate. The surgical site was incised with a sterile scalpel after disinfection with iodine. Operators explored the tumor boundary and completely resected the tumor mass (the resection margin was 3 mm away from the tumor), and then the incision was stitched with absorbable thread. Wound recovery was continuously observed after surgery. After the operation, lung tissues and peripheral blood (PB) were obtained weekly for flow cytometry analyses. Lung tissues were obtained from mice in the 1-week tumor resection group at 2 weeks after surgery and mice in the 2-week tumor resection group at 1 week after surgery for real-time PCR.

### Specimen Acquisition and Processing

All specimens obtained from mice were processed at 9:00 a.m. to prevent the effects of the circadian rhythm. Bone marrow cells were extracted from the hind limbs of mice and filtered. Blood was collected from the eyeball and stored in a heparin-containing tube, followed by lysis for 15 min (BD Bioscience, #349202). The primary tumor and lung tissue were cut into small pieces and digested in medium containing 1 mg/mL collagenase IV (Sigma-Aldrich, #V900893) at 37°C in a constant temperature shaker for 2 h. The cell suspension was then filtered through 40-μm Nylon mesh (BD FALCON, #352340) for subsequent detection or culture.

### Magnetic Isolation of Mouse Neutrophils

The separation of mouse neutrophils was performed according to the isolation protocol provided by the manufacturer (Mouse Neutrophil Isolation Kit, Miltenyi, #130-097-658). Briefly, single cells were obtained from PB, bone marrow (BM) or lung tissue as described above. Fifty microliters of the Neutrophil Biotin-Antibody Cocktail were added as primary antibodies to 200 μL of the cell suspension (5x10^7^ total cells) and incubated for 15 min at 4°C. After washing, 100 µL of Anti-Biotin MicroBeads was added to 400 μL of the cell suspension. An LS column and MidiMACS separator (Miltenyi) were applied for subsequent magnetic sorting.

### Flow Cytometry Analysis and Sorting

The immune cells that had infiltrated the primary tumor, lung, PB and BM were isolated and stained with fluorochrome-conjugated anti-mouse monoclonal Abs specific for CD45 (clone 30-F11), CD11b (clone M1/70), Ly6G (clone 1A8), CD62L (clone MEL-14), CXCR4 (clone L276F12), ICAM-1 (clone YN1/1.7.4), CD11c (clone N418), CD49d (clone R1-2) using standard protocols. The above mentioned mAbs were purchased from Biolegend. For the flow cytometry analysis, data were acquired with a FACS Canto II flow cytometer (BD Biosciences) and analyzed with FlowJo software (V10 for Windows). For flow cytometry sorting, a single-cell suspension was sorted with a FACS Aria III cell sorter (BD Bioscience)

### RNA Isolation and Quantitative Real-Time PCR

Mouse tissues were ground with a ceramic mortar containing liquid nitrogen, and total RNA was extracted from the tissue samples using TRIzol reagent (Invitrogen, #15596-018). Cell samples were added directly to TRIzol according to the manufacturer’s instructions. One microgram of total RNA was reverse transcribed into cDNAs using PrimeScript™ RT Master Mix (TaKaRa, #RR036A), amplified with TB Green Premix Ex Taq (TaKaRa, #RR420A) and detected using the 7500 Fast Real-Time system (Applied Biosystems). Data were processed using 7500 V2.3 software (Applied Biosystems). The results were normalized to the housekeeping gene β-actin and then presented as fold upregulation compared with that of the control.

### Collection of the Tissue Culture Supernatant

Lung tissue obtained from 2-week tumor-bearing mice as described above. Tissues were cut into small pieces using sterile scissors. Then, samples were placed in a 6-well plate with FBS-free RPMI-1640 medium. After 24 h of cultivation, supernatant was harvested and centrifuged at 300x g for 5 min for further purification. The tumor tissue culture supernatant was used for neutrophil induction *in vitro*.

### Metabolic Labeling of Neutrophils Using BrdU

Mice were injected (*i.p.*) with 2.5 mg of BrdU (Thermo Fisher, #000103) (**Figure S3**). Neutrophils containing BrdU that were present in the mouse PB and lung samples were detected using an anti-BrdU antibody. Briefly, single-cell suspensions from PB and lung tissues were first stained for cell surface antigens (CD45-APC/Cy7, CD11b-PE/Cy7, Ly-6G-APC, and CD62L-PE), and then fixed and permeabilized using a BrdU Flow Kit (BD Bioscience, #557891). Cells were treated with DNase I (Sigma-Aldrich, 100 μg/mL, #D5025) for 1 h at 37°C to expose BrdU incorporated in DNA. Then, cells were stained for BrdU with fluorescent antibodies (anti-BrdU-FITC, Biolegend, #364103).

### Neutrophil Adhesion Assay

CD62L^dim^ and CD62L^hi^ neutrophils were obtained from the PB of 4T1 tumor-bearing mice after 1 week *via* single-cell FACS sorting. Then, the sorted cell density was adjusted to 1x10^6^ cells/mL, and cells were seeded on poly-D-lysine-coated slides and incubated for 3 h. Fluorescent yellow-green latex beads (Sigma-Aldrich, #L4655) were added to the wells and gently shaken on the transference shaker. After shaking for 5 min, the wells were gently washed with PBS and cells were observed using a fluorescence microscope. Trypan blue was applied to quench the fluorescence and confirm that the beads had adhered to the neutrophil membrane. After adding Trypan blue, the fluorescence was almost completely quenched suggesting that beads had adhered to the surface of cells rather than being engulfed (data not shown).

### Neutrophil Chemotaxis Assay

CD62L^dim^ and CD62L^hi^ neutrophils were obtained from the PB of 1-week tumor-bearing mice by FACS. For flow cytometry, neutrophil subsets (1x10^5^ cells) were labeled with a fluorescent antibody (Ly-6G-PE or Ly-6G-APC/Cy7), suspended in serum-free culture medium, mixed at a cell ratio of 1:1 and seeded into 24-well Transwell inserts (5.0 μm, Corning) (**Figure S2D**). Medium supplemented with recombinant murine CXCL12 (100 ng/mL, Peprotech, #250-20A), lung tissue culture supernatant (LTCS), or a CXCL12 neutralizing antibody (200 μg/mL, R&D Systems, #MAB310-SP, clone: 79014) was added to the remaining receiver wells. After 1 h, the cells in the wells were collected for flow cytometry.

For the confocal microscopy observation, the wells of the plate were pre-coated with PDL, and the cells were stained with the fluorescent lipid dye-DiO or DiI (Beyotime, #C1038 & #C1036) before being seeded into 24-well Transwell inserts (5.0 μm, Corning). After 1 h, the liquid was aspirated and the wells were gently washed with PBS before confocal microscopy observations.

### *In Vivo* Neutrophil Transfusion Assay

The main experimental design is shown in **Figure S2B**. For the transfusion of a mixture of two cell populations (1:1), neutrophils were isolated from following groups: (1) PB of 3-day tumor-bearing mice after injection of recombinant murine G-CSF (rmG-CSF, 10mg/kg, Peprotech, #250-05); (2) PB of 2-week tumor-bearing mice. The former cells are regarded as CD62L^hi^ neutrophils, and the latter cells are regarded as CD62L^dim^ neutrophils (confirmed by flow cytometry, as shown in **Figure S2C**). Fluorescent antibody labeling (Ly-6G-FITC or Ly-6G-APC) was performed separately, the cell density was adjusted to 1x10^7^ cells/50 μL, the cells were mixed at a 1:1 ratio, and then the cells were injected into the 2-week tumor-bearing mice (*i.v.*). Mouse lung tissue was obtained at the indicated time point (10 min after the injection) and processed into single cells for the flow cytometry analysis.

### Neutrophil Tracking *In Vivo*

Briefly, CD62L^dim^ and CD62L^hi^ neutrophils were obtained as described in the method for the *in vivo* transfusion. The cells were stained with the fluorescent lipid dye DiR (Yeasen, #40757ES25), and then the cell density was adjusted to 1x10^6^ cells/100 μL. The cells were injected into naïve mice or 2-week tumor-bearing mice (female BALB/c or BALB/c nude mice) (*i.v.*), and the DiR signal was detected at the indicated time point (30 min) using an IVIS Lumina LT instrument (PerkinElmer).

### Tissue Hematoxylin and Eosin and Immunofluorescence Staining

Mouse tissues were obtained for hematoxylin and eosin (H&E) staining or immunofluorescence staining and fixed with 4% paraformaldehyde (PFA) for 24 h, followed by embedding by paraffin. Immunofluorescence staining was performed using a standard protocol. Briefly, 4-5-μm paraffin sections were deparaffinized through a gradient of alcohol solutions and rehydrated in water. Antigen retrieval was performed using TRIS-EDTA (pH= 9) buffer in thermostatted bath at 98°C for 30 min. Sections were blocked with 3% BSA (MP Biomedicals) for 60 min. The cells were then incubated with primary antibodies against Ly-6G (Servicebio, #GB11229, 1:200) at 4°C overnight. Next, a Alexa Fluor^®^ 594 Goat Anti-Rabbit secondary antibody (Abcam, #ab150080, 1:500) was added to the sections and incubated at 4°C for 2 h. After washing, DAPI (Invitrogen, Molecular Probes) was added, and the sections were covered with a coverslip. Slides were scanned using Pannoramic MIDI (3DHISTECH Ltd.), and images were captured using Pannoramic Viewer software (3DHISTECH Ltd.).

### Neutrophils Survival *In Vitro* Assay

CD62L^dim^ and CD62L^hi^ neutrophils were obtained from 2-week 4T1 tumor-bearing mice lung *via* single cell FACS sorting. Cell suspension (1x10^5^/100μl complete RPMI-1640 medium) was put into plate bottom 96 wells and harvested per 1 h. Cells were labeled with Annexin V-FITC and PI (Dojindo, #AD10), followed by flow cytometry analysis.

### Macrophage Phagocytosis Assay

For macrophage phagocytosis experiment, lung tissue from 2-week tumor-bearing mice and naive mice as obtained and digested into single cell suspension described above. Then cell suspension was stained with fluorochrome-conjugated anti-mouse monoclonal Abs specific for Biotin anti-mouse F4/80 (clone BM8) at 4°C for 15 min in dark. After washed with cell staining buffer and centrifuged at 300 g for 5 min, MojoSort™ Streptavidin Nanobeads were added into 100μl cell suspension and incubated at 4°C for 15 min in dark. After second wash step and positive magnetic sorting was operated by EasySep Magnets (STEMCELL) according to the manufacturer’s instructions.

Then macrophage was seeded into 12 wells plate and Fluorescent yellow-green Latex beads (Sigma-Aldrich, #L4655) were added in the wells and gently shaking on the Transference Shaker. After 30min, the plate was washed with PBS softly to exclude extra beads and analyzed in LSM 710 confocal microscope (Carl Zeiss, Germany).

### Statistical Analysis

Statistical analyses were performed using GraphPad Prism V6.0 software. The statistical significance (*p ≤ 0.05, **p ≤ 0.01, and ***p ≤ 0.001) of differences in the means between a minimum of three groups was determined using unpaired two-tailed Student’s t test and two-way ANOVA. The results are presented as the mean value ± SD. All results are representative of at least three independent experiments.

## Results

### Neutrophils Participate in the Formation of the Lung Pre-Metastatic Niche for Breast Cancer

**Figure 1:**
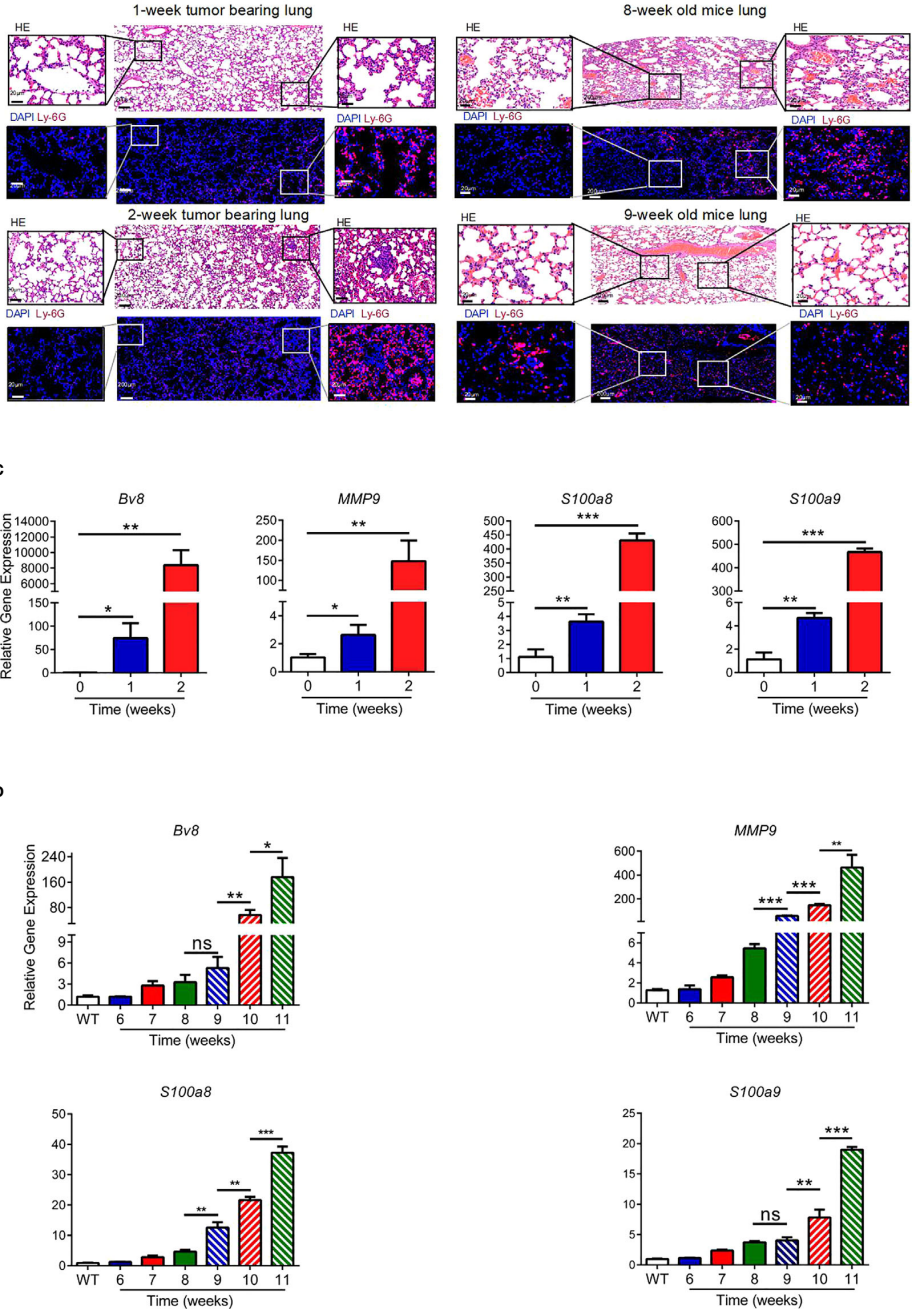
Neutrophils participate in the formation of the lung premetastatic niche for breast cancer. **(A, B)** H&E staining and immunofluorescence staining for neutrophils in continuous sections of the lung tissues (less than 4-μm intervals) from 1- and 2-week tumor-bearing mice **(A)** and 8- and 9-week-old mice in the MMTV-PyMT model **(B)**. **(C**, **D)** The expression of prometastatic genes in the lungs of the 4T1 model at different stages **(C)** and MMTV-PyMT model **(D)**, including *BV8, mmp9, S100a8* and *S100a9*. The data were normalized to expression in naïve mice, as shown in the first column. *β-Actin* was assayed as a control. Data are presented as the mean ± SEM of one representative experiment. Similar results were obtained in three independent experiments, unless indicated otherwise. Unpaired Student’s t tests, ns, not significant. *p < 0.05, **p < 0.01, and ***p < 0.001.

To explore the structural reorganization of the microenvironment of the lung pre-metastatic niche for breast cancer, an orthotopic breast cancer model was established with highly invasive 4T1 tumor cells, and the spontaneous breast cancer model of MMTV-PyMT was generated. No metastasis was detected, but an uneven distribution of infiltrated neutrophils was observed in the lung in the first week after the injection of 4T1 cells into mice. In the second week, lung micrometastases surrounded by large numbers of infiltrating neutrophils and tissue remodeling were observed, while few neutrophils were observed in the nonmetastatic area (**Figure 1A**). Similar results were obtained from the MMTV-PyMT model using 8- and 9-week-old mice (**Figure 1B**). Previous studies with different models have reported diverse time points of pre-metastatic niche formation (18, 37, 38). Lung micrometastases formed at 2 weeks in the 4T1 model and at 9 weeks in the MMTV-PyMT model. Pre-metastatic niche-associated genes, including *Bv8, S100a8, S100a9, and Mmp9*, which were reported to promote tumor cell invasion, migration, and colonization in the metastatic site (18), were upregulated in lung tissues from 1 to 2 weeks in the 4T1 tumor-bearing model (**Figure 1C**) and 9- to 10-week-old mice of the PyMT model (**Figure 1D**) before tumor metastasis. Based on this part of the experiment, we speculated that neutrophils may be involved in the formation of lung pre-metastatic niche of breast cancer.

### Tumor Resection Before Niche Formation Results in a Favorable Prognosis

**Figure 2:**
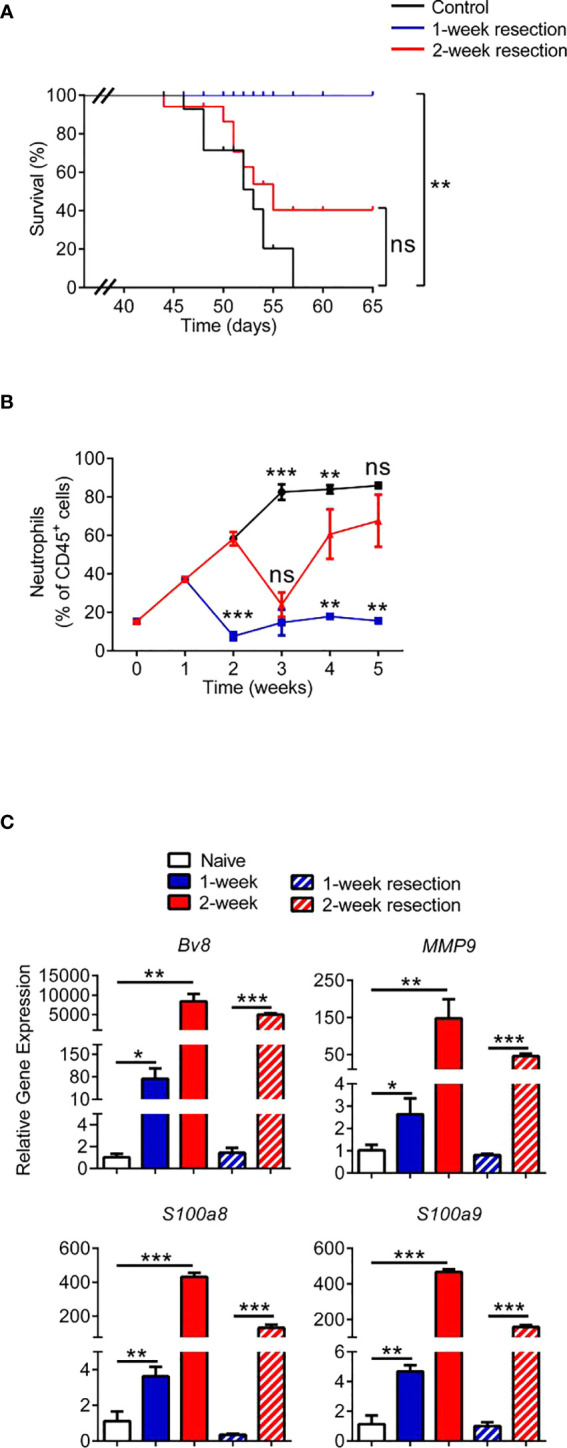
Tumor resection before niche formation results in a favorable prognosis. **(A)** Analysis of the survival of the control group and 1-week or 2-week primary tumor resection groups (n=10 mice per group) (p < 0.001; Kaplan-Meier test). **(B)** Flow cytometry analysis of weekly dynamic changes in lung-infiltrating neutrophils in the control group and 1-week or 2-week primary tumor resection groups (n=3–8 mice per group). **(C)** The expression of prometastatic genes in the lungs of tumor-bearing mice and tumor-resected mice. Data were normalized to expression of naïve mice, as shown in the first column. Data are presented as the mean ± SEM of one representative experiments. Similar results were obtained in three independent experiments, unless indicated otherwise. Unpaired Student’s t tests, ns, not significant. *p < 0.05, **p < 0.01, and ***p < 0.001. See also **Figure S1**.

The primary tumor was resected from the 4T1 tumor-bearing mice at different stages to further accurately define the time period of niche formation (**Figure S1A**). The survival of mice in the 1-week resected group was significantly prolonged (**Figure 2A**), while mice in the 2-week resected group did not exhibit an obvious increase in survival compared with the control group, and mice in both groups died of lung metastasis (**Figure S1B**). Therefore, we considered that the lung pre-metastatic niche had been formed at the second week after 4T1 tumor cell inoculation, resulting in the ineffectiveness of primary tumor excision. Furthermore, the percentage of lung neutrophils gradually returned to normal levels in the 1-week resected group, while the percentage of neutrophils rapidly increased to the level of the nonoperative group after a transient decrease in the 2-week resection group (**Figure 2B**). In addition, niche-associated genes were conspicuously downregulated in the 1-week resected group but not in the 2-week resected group (**Figure 2C**). These findings, together with the data on the pre-metastatic niche obtained at different time points in different models, provide evidence that neutrophils were participants of pre-metastatic niche formation.

### CD62L^dim^ Neutrophils Aggregate in the Lung During Pre-Metastatic Niche Formation

**Figure 3:**
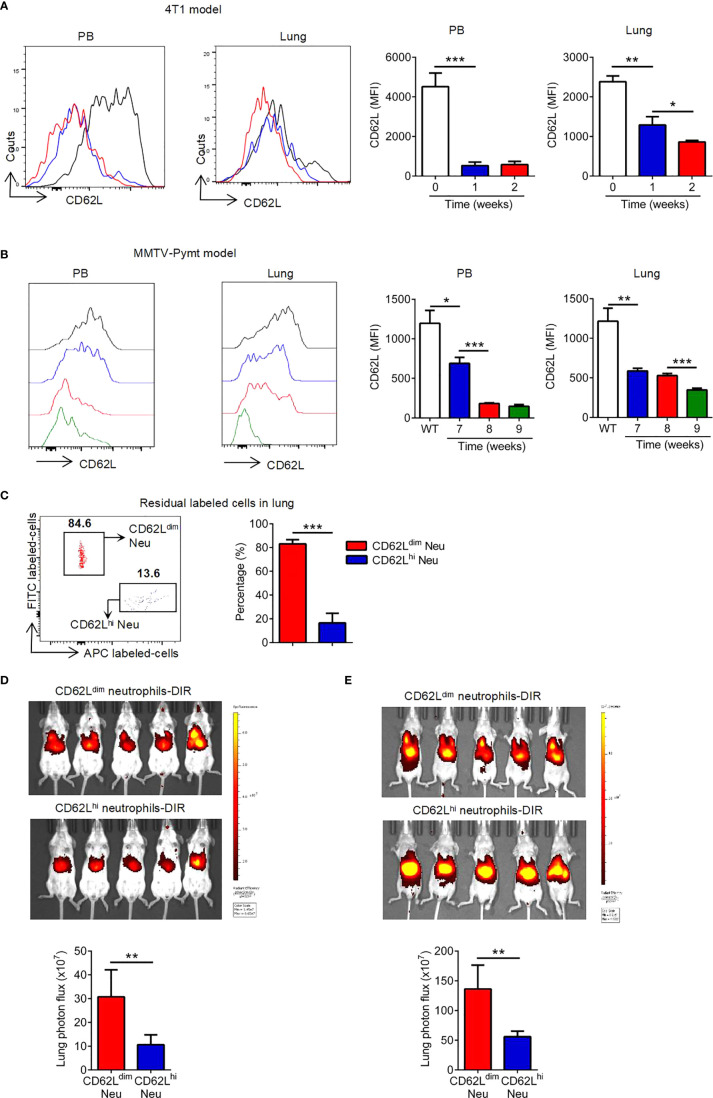
CD62L^dim^ neutrophils aggregate in the lung during premetastatic niche formation. **(A**, **B)** Mean fluorescence intensity (MFI) of CD62L expression on PB and lung-infiltrating neutrophils in tumor-bearing mice from the 4T1 model **(A)** and MMTV-PyMT model **(B)**, as detected using flow cytometry. **(C)** Flow cytometry analysis (left panel) and quantification (right panel) of residual labeled cells in the lungs of 2-week tumor-bearing mice after the reinjection of CD62L^dim^ and CD62L^hi^ neutrophils at a 1:1 ratio. **(D**, **E)** Representative *in vivo* images (up panels) and quantitative analysis of the distribution of DiR-labeled CD62L^dim^ and CD62L^hi^ neutrophils in the lungs (bottom panel) after the tail vein injection into 2-week tumor-bearing BALB/c mice **(D)** and BALB/c nude mice **(E)**. Data are presented as the mean ± SEM of one representative experiment. Similar results were obtained in three independent experiments. Unpaired Student’s t tests, ns, not significant. *p < 0.05, **p < 0.01, and ***p < 0.001. See also **Figure S2**.

A significant increase in the aggregation of CD62L^dim^ neutrophils was observed in PB and lung during the formation of the pre-metastatic niche (**Figures 3A** and **S2A**), similar to the PyMT mice (**Figure 3B**). An *in vivo* transfusion of CD62L^dim^ and CD62L^hi^ neutrophils was performed to investigate the mechanism by which CD62L^dim^ neutrophils accumulate in the lung (**Figures S2B** and **S2C**). The residual fluorescence signal in the lung was primarily derived from CD62L^dim^ neutrophils but not CD62L^hi^ neutrophils (**Figure 3C**), indicating that CD62L^dim^ neutrophils were more likely to accumulate in the lung. DiR-labeled CD62L^dim^ and CD62L^hi^ neutrophils were injected into tumor-bearing mice to confirm and better understand this phenomenon. The signals for CD62L^hi^ neutrophils were mainly distributed in the liver, while signals for CD62L^dim^ neutrophils still partially remained in the lung (**Figure 3D**). A similar result was observed in nude mice (**Figure 3E**), suggesting that this effect did not depend on T cells.

### CD62L^dim^ Neutrophils Initially Aggregate in the Lung by Expressing CXCR4 at High Levels

**Figure 4:**
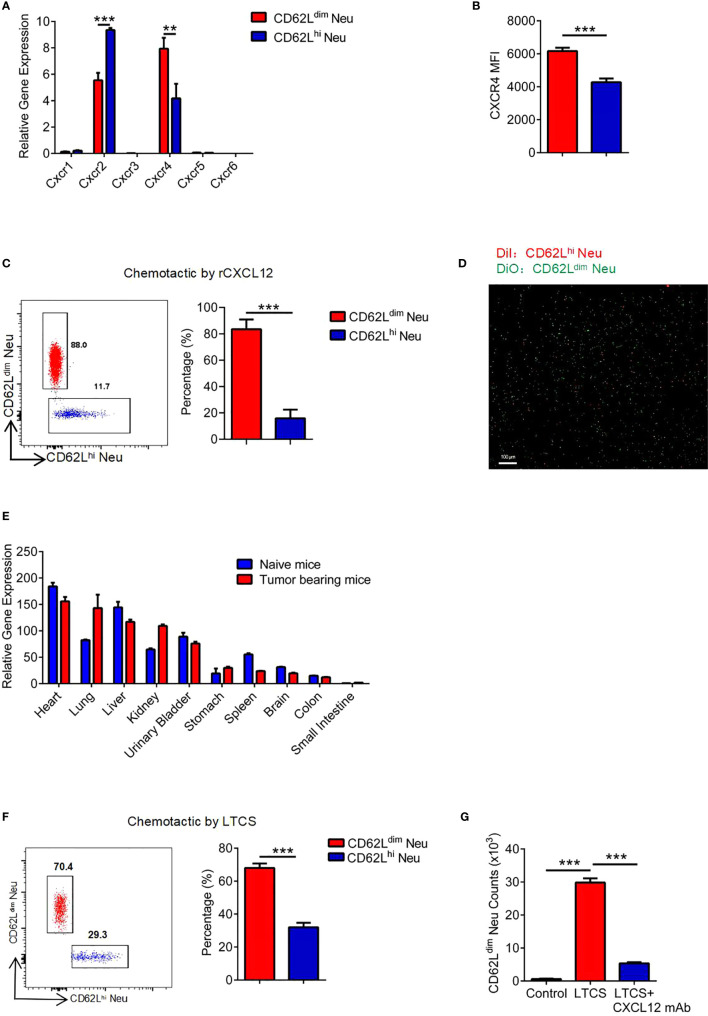
CD62L^dim^ neutrophils initially aggregate in the lung by expressing CXCR4 at high levels, **(A)** RT-PCR analysis of chemokine receptor expression in CD62L^dim^ and CD62L^hi^ neutrophils. *β-Actin* expression in CD62L^hi^ neutrophils was assayed as a control. **(B)** MFI of CXCR4 expression on CD62L^dim^ and CD62L^hi^ neutrophils from tumor-bearing mice, as analyzed using flow cytometry. **(C)** Flow cytometry analysis of the chemotaxis of cells induced by rCXCL12 after a 1:1 mixture of CD62L^dim^ and CD62L^hi^ neutrophils were seeded in the upper chamber. **(D)** Fluorescence imaging of labeled neutrophils after rCXCL12 chemotaxis. **(E)** RT-PCR analysis of *Cxcl12* expression in various organs of naïve mice and 2-week tumor-bearing mice. **(F)** Flow cytometry analysis of the proportion of neutrophils after chemotaxis using the lung tissue culture supernatant (LTCS) of cells from tumor-bearing mice. **(G)** Flow cytometry analysis of the number of CD62L^dim^ neutrophils after chemotaxis using the LTCS of cells from tumor-bearing mice in the presence or absence of the anti-CXCL12 mAb. Data are presented as the mean ± SEM of one representative experiment. Similar results were obtained in three independent experiments. Unpaired Student’s t tests, ns, not significant. *p < 0.05 and ***p < 0.001. See also **Figure S2**.

Neutrophil translocation is regulated by chemokines, allowing them to reach their destinations quickly. Therefore, we measured the expression of chemokine receptors 1 to 6 on neutrophils in the PB of tumor-bearing mice (**Figure 4A**). CXCR4 was expressed at significantly higher levels on CD62L^dim^ neutrophils than on CD62L^hi^ neutrophils (**Figure 4B**), which might explain why the CD62L^dim^ neutrophils trafficked to the lung (39). We further confirmed that CD62L^dim^ neutrophils were more susceptible to chemotaxis mediated by CXCL12 than CD62L^hi^ neutrophils (**Figures 4C, D** and **S2D**). CXCL12 expression in the lung was second only to the heart in the tumor-bearing mice (**Figure 4E**), leading to the chemoattraction of more CD62L^dim^ neutrophils (**Figures 4F** and **S2E**), but these changes were blocked by a CXCL12 mAb (**Figure 4G**).

### CD62L^dim^ Neutrophils Exhibit Stronger Adhesion

**Figure 5:**
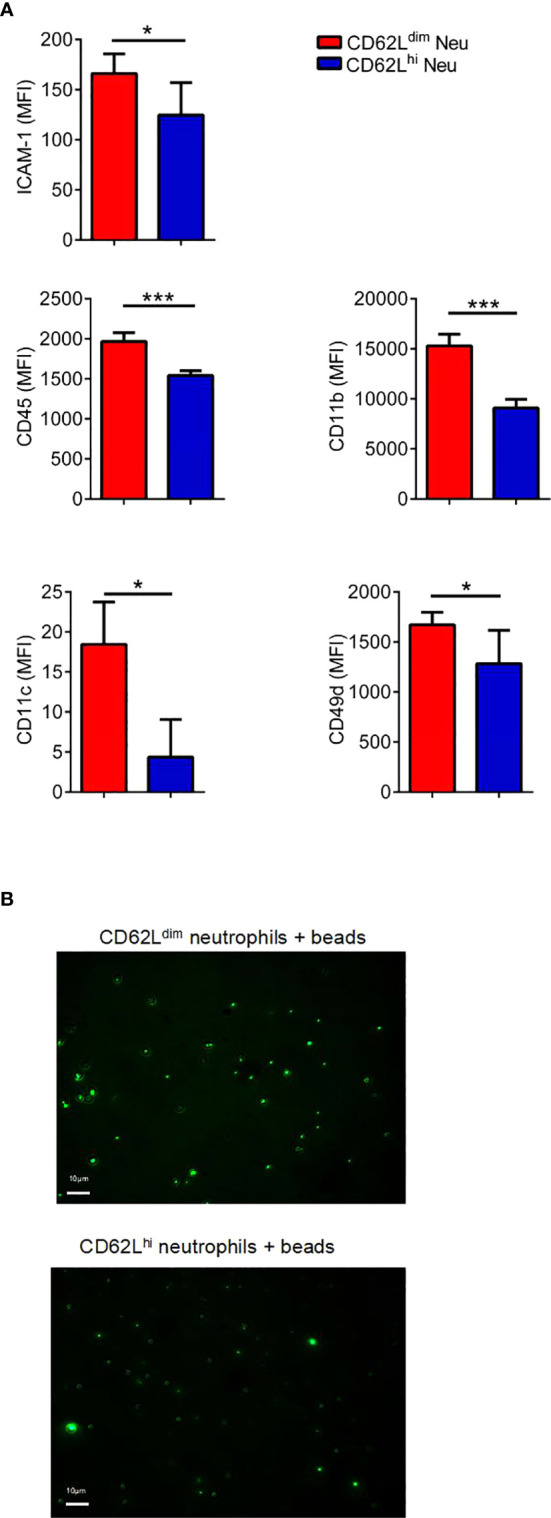
CD62L^dim^ neutrophils exhibit stronger adhesion. **(A)** MFI of adhesion molecules expressed on PB CD62L^dim^ and CD62L^hi^ neutrophils from 1-week tumor-bearing mice, as analyzed using flow cytometry. **(B)** Fluorescence intensity and images of residual fluorescent yellow-green latex beads adhered to CD62L^dim^ and CD62L^hi^ neutrophils. Data are presented as the mean ± SEM of one representative experiment. Similar results were obtained in three independent experiments. Unpaired Student’s t tests, ns, not significant. *p < 0.05 and ***p < 0.001.

In addition to chemokines, the adhesion capacity of circulating neutrophils determines whether they can migrate to the tissue more quickly (40). Our results obtained from 1-week tumor-bearing mice suggested that due to the higher expression of CD45, CD11b, CD11c, CD49d and ICAM-1 on PB CD62L^dim^ neutrophils (**Figure 5A**), the adhesion of CD62L^dim^ neutrophils was stronger than that of CD62L^hi^ neutrophils (**Figure 5B**).

### The Prolonged Survival of CD62L^dim^ Neutrophils Accounts for Their Passive Accumulation in Lung Tissues

**Figure 6:**
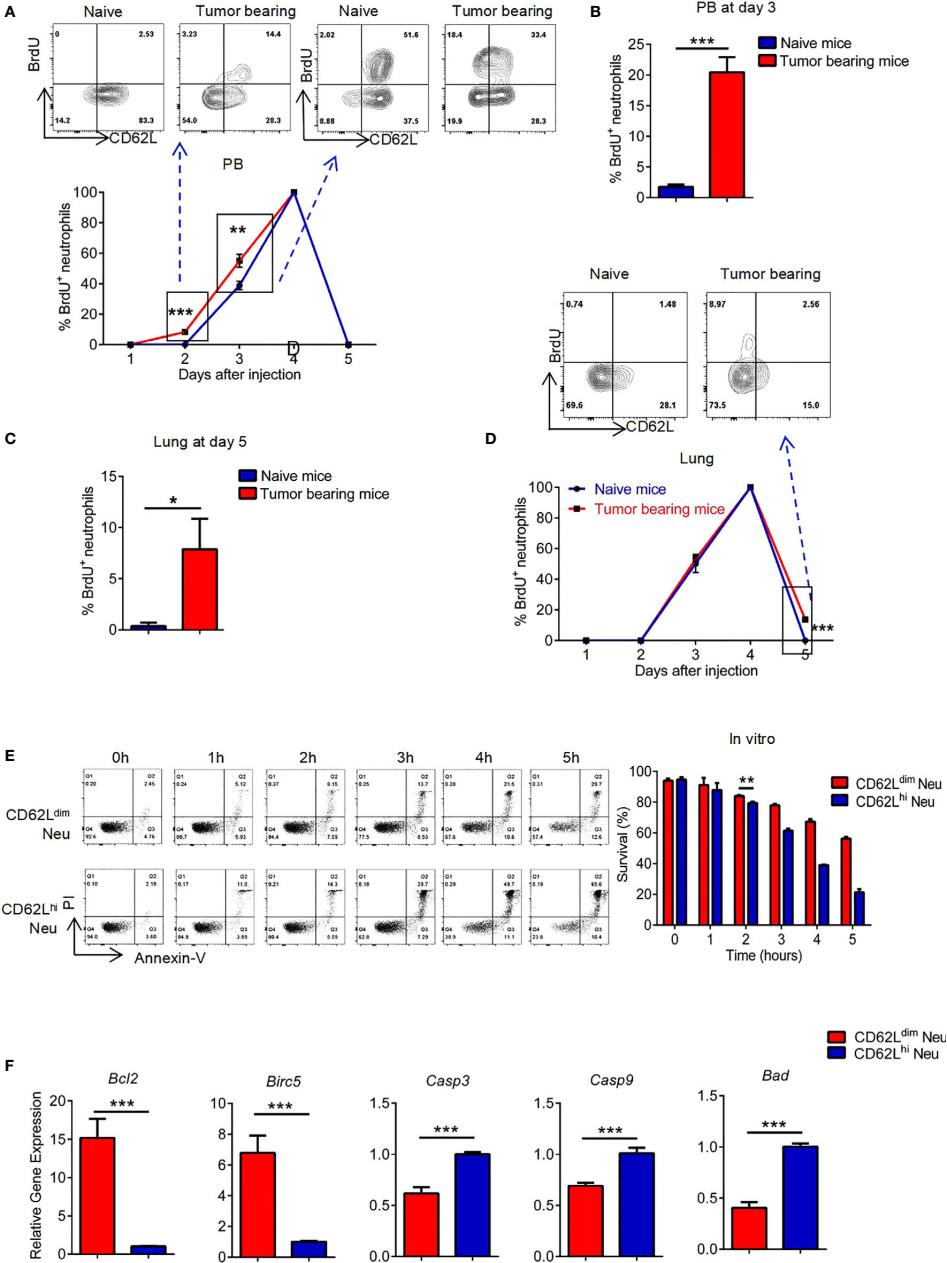
The prolonged survival of CD62L^dim^ neutrophils accounts for their passive accumulation in lung tissues. **(A** to **D)** Flow cytometry analyses of the levels of CD62L and BrdU in neutrophils from the PB **(A**, **B)** and lungs **(C**, **D)** of naïve mice and tumor-bearing mice (gated as CD45^+^CD11b^+^Ly-6G^+^). **(E)** Flow cytometry analysis (left panel) and quantification (right panel) of the ratio of surviving CD62L^dim^ and CD62L^hi^ neutrophils *in vitro*. **(F)** RT-PCR analysis of changes in the expression of apoptosis-related genes in CD62L^dim^ and CD62L^hi^ neutrophils. Data are presented as the mean ± SEM of one representative experiment. Similar results were obtained in three independent experiments. Unpaired Student’s t tests, ns, not significant. *p < 0.05, **p < 0.01, and ***p < 0.001. See also **Figure S3**.

BrdU was used to examine the dynamic disposition of neutrophils leaving the bone marrow (BM) (**Figure S3A**) (41). BrdU^+^ neutrophils appeared in PB of tumor-bearing mice earlier and in greater numbers than in naïve mice, which were mainly CD62L^hi^ neutrophils (**Figure 6A**, left panel). Three days after the BrdU injection, however, those BrdU^+^ neutrophils in tumor-bearing mice quickly transformed from CD62L^hi^ neutrophils to CD62L^dim^ neutrophils, while neutrophils in naïve mice were still CD62L^hi^ neutrophils (**Figures 6A**, right panel, and **6B**). BrdU^+^ neutrophils were still detected in greater numbers in the lung tissues of tumor-bearing mice than in naïve mice on the fifth day and were all CD62L^dim^ neutrophils (**Figures 6C, D****)**. Based on these results, CD62L^dim^ neutrophils might persist for longer periods in lung tissues. In support of our hypothesis, CD62L^dim^ neutrophils survived for longer periods than CD62L^hi^ neutrophils *in vitro* (**Figure 6E**), and in total neutrophils we found that the percentage of CD62L^dim^ neutrophils gradually increased over time (**Figure S3B**); meanwhile, the CD62L^dim^ and CD62L^hi^ neutrophils never interconverted without stimulation *in vitro* (**Figure S3C**). In addition, the expression of antiapoptotic genes was upregulated in CD62L^dim^ neutrophils, but the expression of proapoptotic genes was reduced (**Figure 6F**), consistent with the prolonged survival of CD62L^dim^ neutrophils. The clearance of neutrophils in lung tissue is mainly mediated by macrophages (42). In our study, the ability of macrophages from tumor-bearing mice compared to cells from naïve mice to engulf FITC beads was significantly reduced (**Figure S3D**), which might explain why CD62L^dim^ neutrophils were slowly cleared from the lung. Collectively, CD62L^dim^ neutrophils exhibited greater chemotaxis to the lung tissue, stronger adhesion, and longer survival without clearance, inducing continuous recruitment to the lung tissue and the subsequent formation of the pre-metastatic niche.

## Discussion

The pre-metastatic niche in distant organs is a specific site created for tumor colonization and growth and is considered a crucial stage of tumor metastasis and an important cause of metastasis to specific organs. However, the exact time when the pre-metastatic niche is formed during tumor progression continues to be debated. A previous study considered the fifth week after tumor inoculation as the niche formation period in a mouse model of 4T1 breast cancer (18), but we observed lung micrometastases and obvious changes in the expression of metastasis-related genes during the second week in the same model. In addition, lung metastasis still developed, despite the removal of the primary tumor at 2 weeks. Therefore, 1 to 2 weeks are believed to be a crucial period for niche formation in the 4T1 tumor model. Similar results revealed that 8 to 9 weeks are the key period for niche formation in the MMTV-PyMT spontaneous breast cancer model.

Neutrophils mature and exit from the BM in homeostasis, then age and are subsequently cleared by local macrophages or return to the BM to maintain a stable population of myeloid cells. In addition to the BM, organs such as the lung, liver, and spleen are also peripheral reservoirs of neutrophils (43), which ensure that neutrophils are able to mount a quick response to inflammatory factors and cytokines when inflammation occurs. Various studies have revealed the effect of neutrophils on promoting tumor metastasis in lung, but a consensus regarding the underlying mechanisms has not yet been achieved (18, 23, 44). Neutrophils can promote metastasis by secreting angiogenic related factors such as VEGFα, and transform extracellular matrix by producing metalloproteinases such as MMP9. Moreover, neutrophils exert a series of immunosuppressive functions similar to PMN-MDSCs that inhibit the proliferation of effector CD8^+^ T cells and its IFN-γ secretions, also inhibit the killing function of NK cells (22, 26). Moreover, neutrophils can also secrete IL-16 and transferrin to accelerate metastasis (45, 46) and remain stemness of tumor cells. Previous studies reveals that exosomes secreted by tumor cells promote neutrophil NETs formation and thus enhance their ability to capture tumor cells (23), while *in vivo* neutrophil depletion by Ly-6G antibody in mouse models had contradictory outcomes (22, 47). These conflict results are thought to be a result of neutrophil heterogeneity (48). In our models, the distribution of neutrophil subsets in lung tissues was already altered in the pre-metastatic niche of breast cancer. Thus, neutrophils in the lung pre-metastatic niche should not be considered a homogeneous group. CD62L^dim^ neutrophils, a subset of granulocytes that have never been reported to be involved in tumor metastasis, accumulated in lung in significant numbers during niche formation, and we discussed the causes of their accumulation.

A number of cytokines have been found to chemoattract and recruit neutrophils to local tissues. It has also been reported that γδT cells secreted IL-17A, obesity induced GM-CSF and IL-5, periostin and Elf5 are associated with neutrophils recruitment in the lung (22, 49–51). Meanwhile, G-CSF is also an important factor to recruit neutrophils (52, 53) and previous studies have found that highly metastatic tumor cell lines, such as 4T1 or 231, secrete more G-CSF than those with low metastatic potential, which is positively correlated with the infiltrating neutrophils, suggesting its important role in tumor metastasis (54, 55). Besides, chemokines are a critical factor contributing to the recruitment of immune cells, particularly the CXCL1/CXCR2 and CXCR4/CXCL12 axes, which are obviously correlated with the aggregation of neutrophils in the lung (39, 56). However, we reported that CXCL12 expression was significantly increased in the lung of the mouse breast cancer model, which recruited a large number of CD62L^dim^ neutrophils through the CXCR4/CXCL12 axis. The expression of CXCR4 on neutrophils increases gradually with the loss of CD62L, which was speculated to explain the importance of CXCR4/CXCL12 signaling in CD62L^dim^ neutrophils (40).

CD62L, a member of the selectin family, plays a vital role in the movement of neutrophils. The adhesion of CD62L^dim^ neutrophils has been suggested to be weaker than CD62L^hi^ neutrophils (57), but another study reported stronger adhesion of CD62L^dim^ neutrophils (40). Furthermore, CD62L^dim^ neutrophils in the pre-metastatic niche of breast cancer, although they have lost L-selectin expression, express other adhesion molecules at higher levels and exhibit increased adhesion, which promote their accumulation in the lung.

In contrast to previous studies considering CD62L^dim^ neutrophils as apoptotic cells (58), our experiment confirmed that CD62L^dim^ neutrophils survived for markedly longer periods than CD62L^hi^ neutrophils both *in vivo* and *in vitro* due to the decreased expression of proapoptotic factors and the increased expression of anti-apoptotic factors. In addition, the functional neutrophils tend to be engulfed locally by macrophages (59). The phagocytosis capacity of macrophages decreased during niche formation. This result may also explain the passive prolongation of the survival of CD62L^dim^ neutrophils. However, the factors that influence macrophage function deserve further investigation.

CD62L^dim^ neutrophils have unique transcriptional characteristics and a greater ability to produce NETs and ROS during inflammation (41, 60); however, other scholars have proposed that these cells are a group of neutrophils with a very weak killing activity (42). Moreover, these cells have been shown to exhibit powerful immunosuppressive function (57, 61). The NETs of neutrophils maintain the residence of metastatic tumor cells in characteristic organs (17, 62), and an immunosuppressive microenvironment may promote the continuous growth of tumor cells in tissues (22). These findings may explain why CD62L^dim^ neutrophils promote tumor metastasis, which deserves further investigation and hence provides guidance for clinical predictions and treatment.

## Data Availability Statement

The datasets generated for this study are available on request to the corresponding author.

## Ethics Statement

The animal study was reviewed and approved by the Ethics Committee of the Second Affiliated Hospital of Zhejiang University School of Medicine in accordance with the Declaration of Helsinki.

## Author Contributions

ZW and CY developed the concept and wrote the manuscript. ZW, CY, and LL designed the experiments. ZW, CY, WC, JL, and FQ performed the experiments, organized the data, and conducted the statistical analyses. ZZ, JP, and KS participated in analyzing and discussing the results. JH conceived the study, participated in its design and coordination, helped draft the manuscript, and supervised the study. All authors contributed to the article and approved the submitted version.

## Funding

This study was supported by grants from the National Natural Science Foundation of China (81872317 to JH, 81520108024 to JH, 81902981 to ZW, and 81672802 to FQ) and the Zhejiang Provincial Natural Science Foundation of China (LZ17H160004 to FQ).

## Conflict of Interest

The authors declare that the research was conducted in the absence of any commercial or financial relationships that could be construed as a potential conflict of interest.

## Supplementary Material

Supplementary Figure 1 related to **Figure 2** **(A)** Schematic illustrating the surgical resection of the primary tumor from the 4T1 model at different stages of tumor progression. **(B)** Anatomy of tumor metastasis in lung tissues from the different groups at 6 weeks (the white arrows indicate metastasis).

Supplementary Figure 2 related to **Figures 3** and **4** **(A)** Flow cytometry gating strategy for the detection of lung-infiltrating neutrophils. **(B** and **C)** Schematic illustrating the strategies used to acquire CD62L^dim^ and CD62L^hi^ neutrophils and for the reinjection of a 1:1 cell mixture in mice **(B)**. Further verification of the purity, properties (gated as CD45^+^CD11b^+^Ly-6G^+^) of the reinjected cells and analysis of the lung after the injection **(C)**. **(D)** Schematic illustrating the analysis of a 1:1 mixture of labeled CD62L^dim^ and CD62L^hi^ neutrophils using the rCXCL12 Transwell chemotactic system. The flow cytometry analysis of the proportion of neutrophils before and after rCXCL12 chemotaxis is also shown. **(E)** Schematic illustrating the analysis of a 1:1 mixture of labeled CD62L^dim^ and CD62L^hi^ neutrophils using the Transwell chemotactic system and LTCS of cells from 2-week tumor-bearing mice.

Supplementary Figure 3 related to **Figure 6** **(A)** Schematic illustrating the BrdU injection into naïve mice and 1-week tumor-bearing mice. **(B)** Flow cytometry analysis (left panel) and quantification (right panel) of the proportion of CD62L^dim^ neutrophils among total lung neutrophils observed each hour in the *in vitro* culture system. **(C)** Flow cytometry analysis of the phenotypic changes before and after the *in vitro* culture of CD62L^dim^ and CD62L^hi^ neutrophils (gated as CD45^+^CD11b^+^Ly6G^+^). **(D)** Flow cytometry analysis (left panel) and quantification (right panel) of the ability of lung macrophages from naïve mice and 2-week tumor-bearing mice to phagocytose yellow-green latex beads.

## Abbreviations

*i.p.*, intraperitoneal; *i.v.*, intravenous; BrdU, 5-bromo-2’-deoxyuridine; BM, bone marrow; PB, peripheral blood; H&E, hematoxylin and eosin; mAb, monoclonal antibody; FBS, fetal bovine serum; LTCS, lung tissue culture supernatant.
