# Supplementary figures and images for: CD62Ldim Neutrophils Specifically Migrate to the Lung and Participate in the Formation of the Pre-Metastatic Niche of Breast Cancer
Source: Front Oncol. 2020 Oct 15;10:540484. doi: 10.3389/fonc.2020.540484 (PMC7593663; doi:10.3389/fonc.2020.540484)

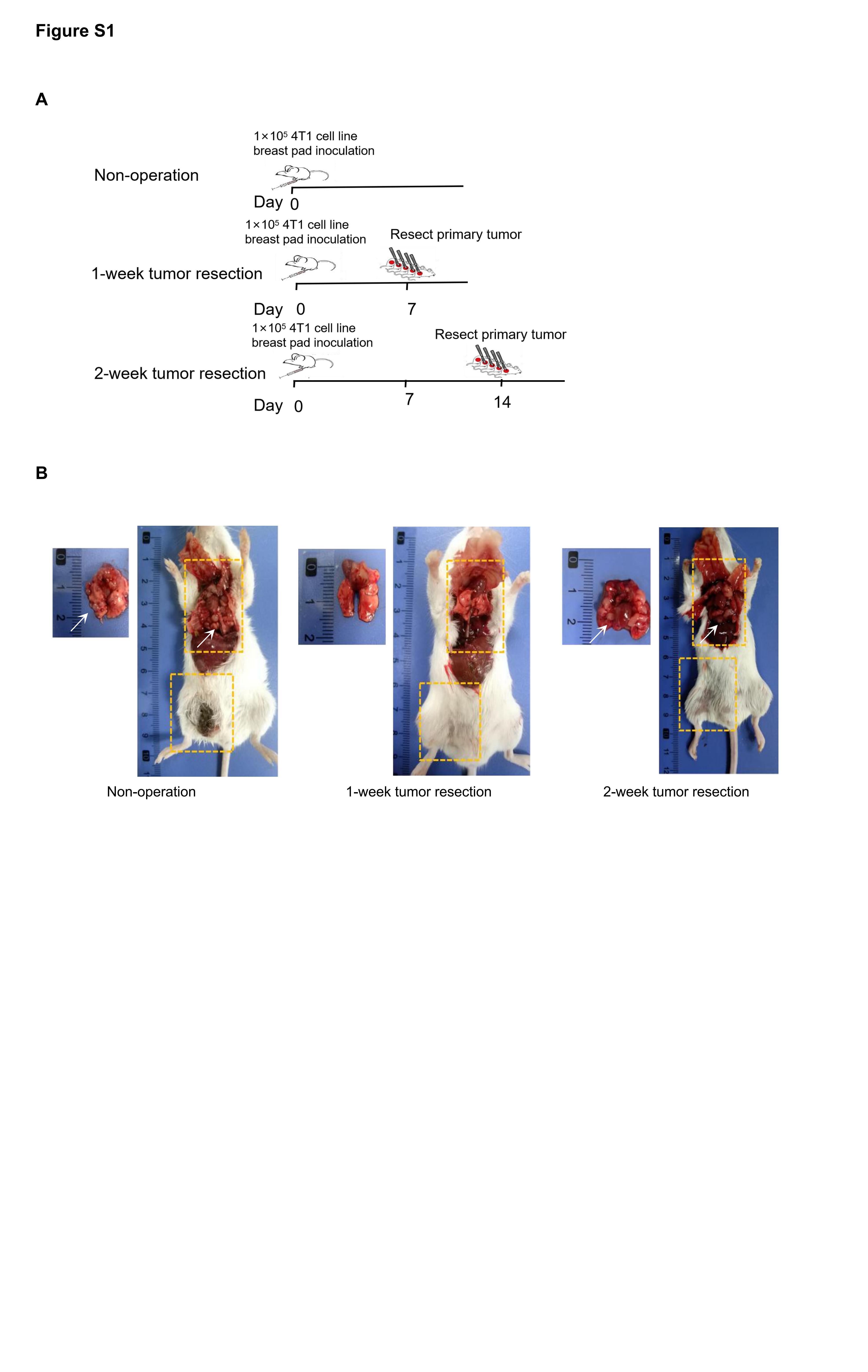

Supplement: Supplementary Figure 1 — related to Figure 2 (A) Schematic illustrating the surgical resection of the primary tumor from the 4T1 model at different stages of tumor progression. (B) Anatomy of tumor metastasis in lung tissues from the different groups at 6 weeks (the white arrows indicate metastasis). [file Image_1.jpeg]

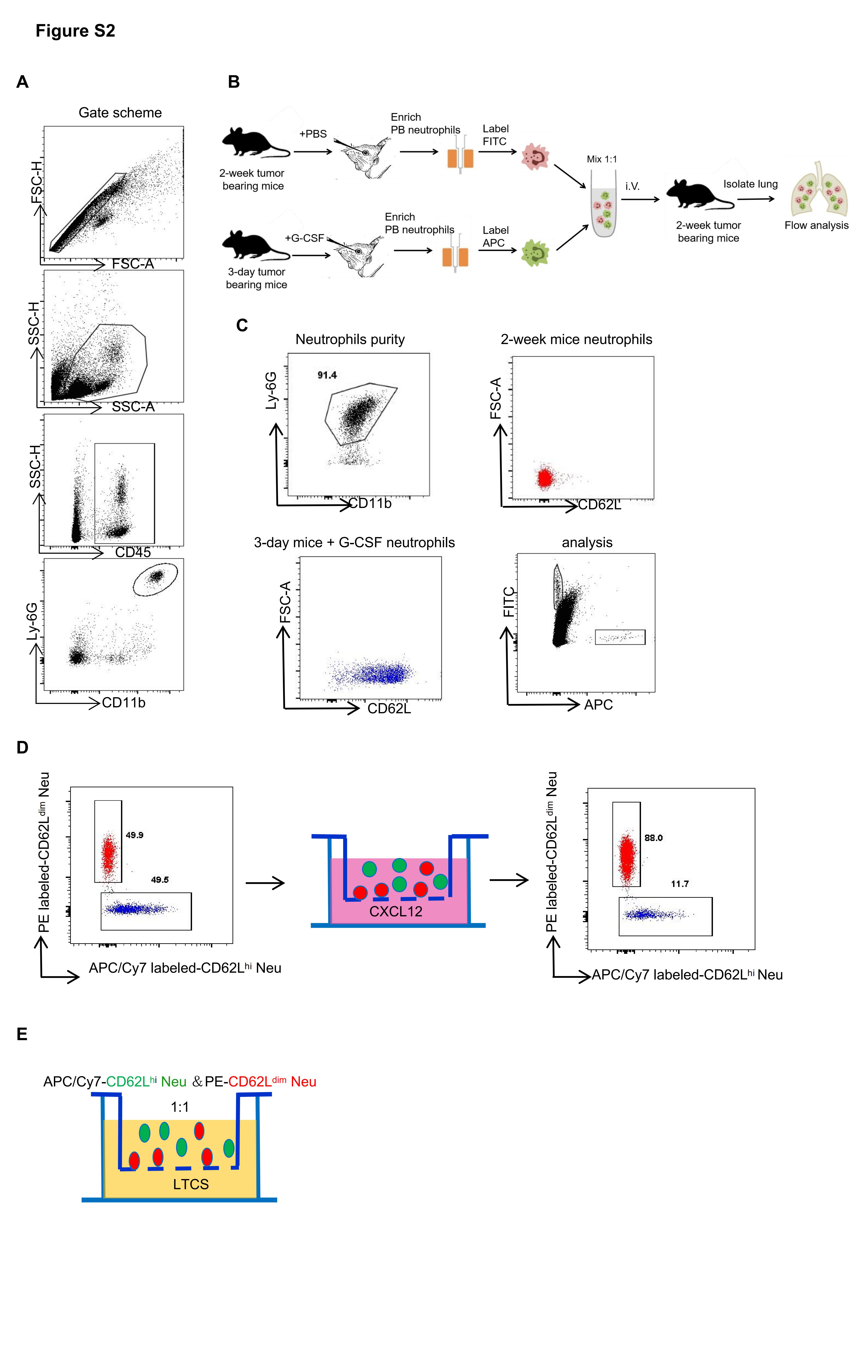

Supplement: Supplementary Figure 2 — related to Figures 3 and 4 (A) Flow cytometry gating strategy for the detection of lung-infiltrating neutrophils. (B and C) Schematic illustrating the strategies used to acquire CD62Ldim and CD62Lhi neutrophils and for the reinjection of a 1:1 cell mixture in mice (B). Further verification of the purity, properties (gated as CD45+CD11b+Ly-6G+) of the reinjected cells and analysis of the lung after the injection (C). (D) Schematic illustrating the analysis of a 1:1 mixture of labeled CD62Ldim and CD62Lhi neutrophils using the rCXCL12 Transwell chemotactic system. The flow cytometry analysis of the proportion of neutrophils before and after rCXCL12 chemotaxis is also shown. (E) Schematic illustrating the analysis of a 1:1 mixture of labeled CD62Ldim and CD62Lhi neutrophils using the Transwell chemotactic system and LTCS of cells from 2-week tumor-bearing mice. [file Image_2.jpeg]

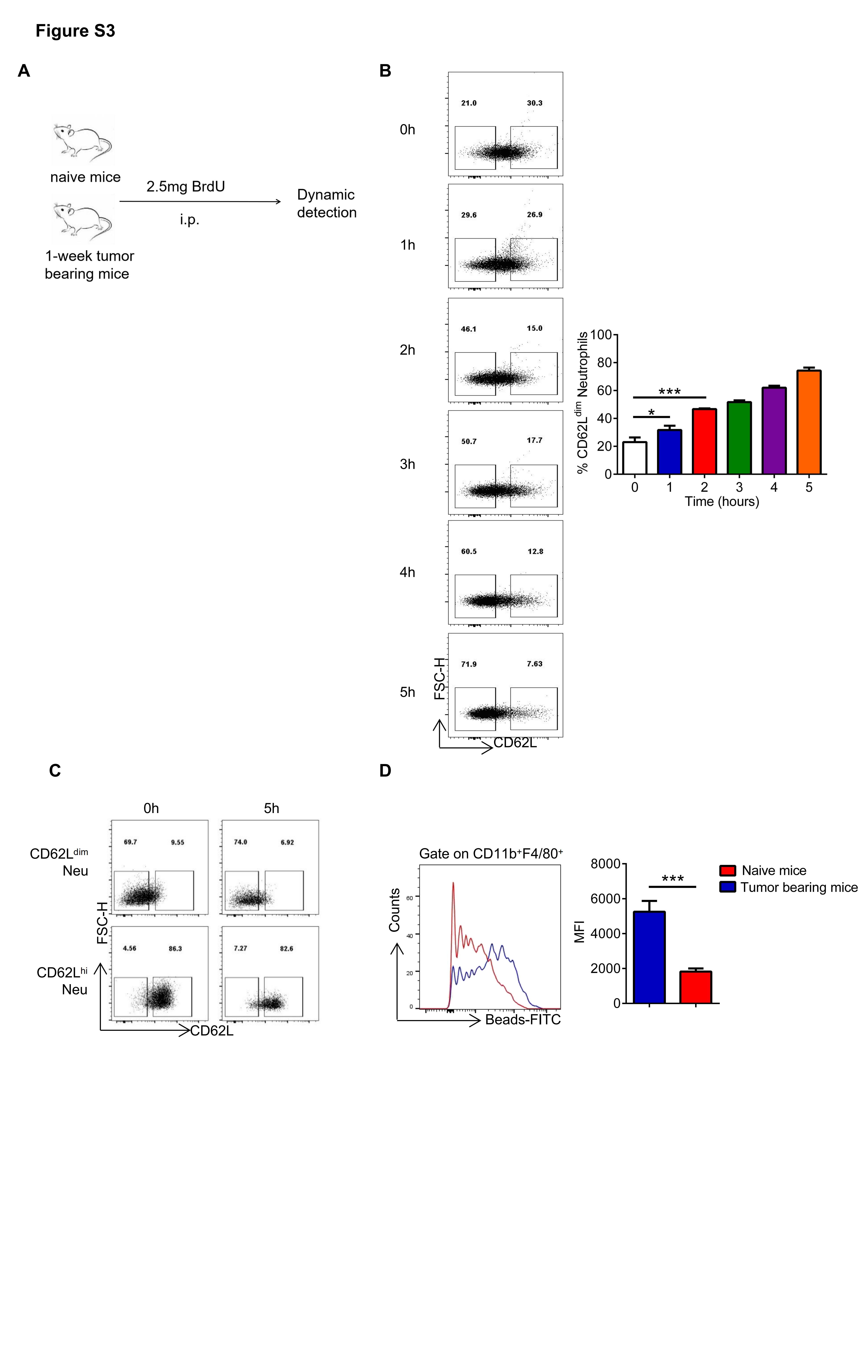

Supplement: Supplementary Figure 3 — related to Figure 6 (A) Schematic illustrating the BrdU injection into naïve mice and 1-week tumor-bearing mice. (B) Flow cytometry analysis (left panel) and quantification (right panel) of the proportion of CD62Ldim neutrophils among total lung neutrophils observed each hour in the in vitro culture system. (C) Flow cytometry analysis of the phenotypic changes before and after the in vitro culture of CD62Ldim and CD62Lhi neutrophils (gated as CD45+CD11b+Ly6G+). (D) Flow cytometry analysis (left panel) and quantification (right panel) of the ability of lung macrophages from naïve mice and 2-week tumor-bearing mice to phagocytose yellow-green latex beads. [file Image_3.jpeg]
